# The Characteristics of Wild Rat (*Rattus* spp.) Populations from an Inner-City Neighborhood with a Focus on Factors Critical to the Understanding of Rat-Associated Zoonoses

**DOI:** 10.1371/journal.pone.0091654

**Published:** 2014-03-19

**Authors:** Chelsea G. Himsworth, Claire M. Jardine, Kirbee L. Parsons, Alice Y. T. Feng, David M. Patrick

**Affiliations:** 1 School of Population and Public Health, University of British Columbia, Vancouver, British Columbia, Canada; 2 Animal Health Centre, British Columbia Ministry of Agriculture, Abbotsford, British Columbia, Canada; 3 Department of Pathobiology, University of Guelph, Guelph, Ontario, Canada; Metabiota, United States of America

## Abstract

Norway and black rats (*Rattus norvegicus* and *Rattus rattus*) are among the most ubiquitous urban wildlife species and are the source of a number of zoonotic diseases responsible for significant human morbidity and mortality in cities around the world. Rodent ecology is a primary determinant of the dynamics of zoonotic pathogens in rodent populations and the risk of pathogen transmission to people, yet many studies of rat-associated zoonoses do not account for the ecological characteristics of urban rat populations. This hinders the development of an in-depth understanding of the ecology of rat-associated zoonoses, limits comparability among studies, and can lead to erroneous conclusions. We conducted a year-long trapping-removal study to describe the ecological characteristics of urban rat populations in an inner-city neighborhood of Vancouver, Canada. The study focused on factors that might influence the ecology of zoonotic pathogens in these populations and/or our understanding of that ecology. We found that rat population density varied remarkably over short geographical distances, which could explain observed spatial distributions of rat-associated zoonoses and have implications for sampling and data analysis during research and surveillance. Season appeared to influence rat population composition even within the urban environment, which could cause temporal variation in pathogen prevalence. Body mass and bite wounds, which are often used in epidemiologic analyses as simple proxies for age and aggression, were shown to be more complex than previously thought. Finally, we found that factors associated with trapping can determine the size and composition of sampled rat population, and thus influence inferences made about the source population. These findings may help guide future studies of rats and rat-associated zoonoses.

## Introduction

Norway and black rats (*Rattus norvegicus* and *Rattus rattus*) are commensal rodents with a virtually worldwide distribution [1]. They are highly adapted to coexisting with human populations, and are particularly ubiquitous in the urban environment [1]–[3].

Infestations are problematic in urban settings because rats are the source of a number of zoonotic pathogens (pathogens transmissible from animals to people) responsible for significant human morbidity and mortality in cities around the world [4], These pathogens include *Leptospira interrogans*, *Rickettsia typhi*, *Yersinia pestis*, *Streptobacillus monilliformis*, and Seoul hantavirus, among others [4]. Urban rat infestations and rat-associated public health risks have been shown to increase in association with the growth of cities and increased urban poverty [4], [5]. Given current unprecedented rates of global urbanization (over half the global population now resides in urban centers [6]), rat-related disease issues are likely to increase in the future.

Rodent ecology is a primary determinant of zoonotic pathogen dynamics in rodent populations and of the risk of pathogen transmission from rats and people [4], [7]. Yet many studies of rat-associated zoonoses (RAZ) do not account for the characteristics of rat populations during sampling or data analysis [8]–[12]. This is problematic because it hinders the development of a comprehensive understanding of pathogen ecology in rat populations, prevents comparability and synthesis of results generated by different studies, and can lead to erroneous conclusions with regard to RAZ dynamics.

The characteristics of rat populations can influence the ecology of RAZ and/or our understanding of RAZ ecology in a variety of ways. For example, the prevalence of many zoonotic pathogens among rats can vary remarkably even over a short geographic distance [13], [14]. The reasons for this are unclear but could be related to geographic variations in rat population density, independent of, or in combination with, environmental factors. Although past research has indicated that urban rats form tight colonies with small home ranges [15], there are very little data regarding how rats are distributed across the modern urban landscape.

The ecology of RAZ could also be influenced by temporal factors, particularly season. Seasonal changes in rodent population dynamics have been shown to influence the ecology of rodent-borne zoonotic pathogens in more sylvatic settings [16], [17]. However, there is considerable conflict in the literature regarding the impact of season on rats and RAZ in urban ecosystems [18]–[21], which, being largely under human control, would seem less prone to natural seasonal variations.

In addition to extrinsic factors, the characteristics of rats themselves can influence the likelihood of infection with a RAZ. Body mass is one of the most common variables used to analyze and interpret data on RAZ, with mass being used as a proxy for chronological age [13], [19], [22], [23]. However, there are a variety of factors other than age that could influence body mass, such as population of origin [24], [25], health status [26], and access to resources [21], [27], [28]. The factors contributing to body mass in urban rats have not been well described.

Rat behavior and social interactions are also likely to have significant impacts on disease transmission but are very difficult to capture and quantify during field research. One aspect of behavior that can be identified is intra-specific aggression resulting in visible cutaneous bite wounds. Past research has identified a positive correlation between Seoul hantavirus infection and the presence of cutaneous bite wounds in urban rats [29]. However, it is difficult to say whether this indicates that the virus is transmitted through biting, or whether bite wounds are a reflection of some underlying characteristic of the individual, behavioral or physiologic, which is driving infection. As with body mass, the factors affecting wounding in urban rats have not been fully investigated.

Finally, most studies of RAZ use data generated though trapping to make inferences about population and disease ecology [18], [19], [30]. These inferences are based on the assumption that all animals in a population are equally likely to enter any given trap at any given time (i.e., that trapping itself does not create any systematic bias in the characteristics of the study population when compared to the source population). However, there are a variety of factors that could influence if and when an animal enters a trap, including trapping methodology, trap environment, and characteristics of the individual rat [27]. Given marked variation among studies with regard to how trapping is carried out [19], [22], [31], [32], it is important to better understand how trapping might influence the number and characteristics of rats trapped.

The overall objective of this study was to describe the characteristics of rat populations from an inner-city neighborhood of Vancouver, Canada, with a focus on factors that could influence the ecology of zoonotic pathogens in these populations and/or our understanding of pathogen ecology. Specifically, we aimed to: 1) Describe rat population density and distribution across the study area; 2) Describe seasonal variations in rat population size and structure; 3) Identify the factors that contribute to body mass in urban rats; 4) Identify the factors that influence the presence and number of cutaneous bite wound in rats; and 5) Describe how factors associated with trapping can influence the success of rat collection and the characteristics of the rat population collected.

## Methods

### Ethics Statement

This study was approved by the University of British Columbia’s Animal Care Committee (A11-0087) and adhered to national guidelines set out by the Canadian Council on Animal Care (www.ccac.ca), including those pertaining to animal user training, euthanasia, protocol review, and wildlife (http://www.ccac.ca/en_/standards/guidelines). This study did not involve any endangered or protected species.

### Study Area

The study was conducted in Vancouver’s Downtown Eastside (DTES) (N49°17′/W123°6′) and included 43 contiguous city blocks covering an area of approximately 82 ha. Also included was 1 private property (3 ha) within an international shipping port at the northern border of the study area. We selected the DTES as the study site because the age and low socioeconomic status of this inner-city neighbourhood [33] produces an environment that may support larger rat populations compared to other areas of the city (i.e., due to building disrepair, accumulated waste, etc.) [4], [34]. Additionally, poverty. as well as concurrent health issues and behaviours, such as injection drug use in public spaces and HIV/AIDS [33], could make DTES residents more vulnerable to rat-associated health risks compared to the general population [4] – suggesting that this area should be a priority for study from a social justice standpoint.

Within the city blocks, trapping took place on public property only and no specific permissions were required. The port site was a private property that wishes to remain anonymous. Permission to trap at this site was obtained from the property manager.

### Trapping Methodology

This study formed the base of a larger project characterizing the ecology of zoonotic pathogens in rat populations. For that project we needed whole rat carcasses for disease analysis, so non-lethal methods, such as mark-recapture, could not be used. We therefore used trapping-removal methods.

Initially, it was hoped that population size could be estimated by tracking decline in catch-per-unit effort [35], [36]. However, after attempting to use this method in the first five blocks under study, we found that it was only effective in city blocks with very large rat populations. More importantly, the prolonged period of trapping activity drew unwanted attention from the public, leading to significant equipment vandalism. For this reason, we elected to switch to a trap success index to measure relative rat abundance among blocks using shorter trapping periods and fewer traps. This method is based on calculating the total number of rats trapped compared to the total number of traps set [35]–[37]. Although trap success methods do not allow calculation of absolute population size, we were more interested in comparisons among blocks, therefore an index of relative rat abundance was considered sufficient.

Each city block and the port site was assigned to a randomly selected 3-week study period over the course of 1 year (September 2011– August 2012), in order to capture seasonal variations in rat ecology [37]. Within each block, approximately 20 Tomahawk Rigid Traps for rats (Tomahawk Live Trap_._, Hazlelhurst, WI) were set out along each side of the back alley that bisected the block. After several episodes of vandalism which involved crushing traps, stainless steel trap covers were designed. These trap covers were locked to the trap and to a chain that secured the trap and cover to an immovable object in order to prevent theft. Traps were evenly spaced where possible, but had to be placed on public property in a location where they did not obstruct traffic and could be secured. Additionally, given that rats prefer to travel along solid surfaces [28], traps were placed alongside a wall whenever possible. Where this was not possible, traps were placed against the nearest solid object (e.g., a fence or dumpster).

For the private property at the international shipping port, 56 traps were placed in 8 locations, which included both indoor and outdoor areas where site employees had seen rats. Also within this property, rats trapped by a private pest control professional in areas not accessible to researchers were also collected.

Traps were pre-baited (filled with bait but fixed open) for 1 week in order to ensure rats were acclimatized to the traps and bait prior to the onset of trapping. Pre-baiting was followed by active trapping for 2 consecutive weeks. Traps were set at 4∶00 pm and checked at 9∶00 am the following day. Traps were not set during the day in order to reduce interactions between rats/traps and people during the period of time when the alleys are most used by area residents. Trapping was conducted on weekdays and traps were baited but fixed open on week-ends. Baits used included combinations of peanut butter, bacon fat, oats, and flour, which were rolled into balls and frozen for easier handling. Fresh apple slices were provided as a water source.

Trapped rats were transferred from the trap to a rodent inhalation narcosis chamber (Sciencelab.com Inc., Houston, Texas) where they underwent isoflurane anesthesia, followed by blood collection via intracardiac puncture and intracardiac pentobarbital euthanasia. All rodent procedures were conducted in the back of a study van in order to protect the researchers and rats, and to prevent public disturbance. Rats collected by the private pest control professional at the port were trapped using snap-type kill traps.

### Data Collection

Georeferenced aerial photographs were used to identify and map the location of each trap within the study area using ArcGIS 10.0 (ESRI, Redlands, USA). For each trap, the number of rats caught in that trap and the proximity of the trap to the nearest building (i.e., directly against a solid wall vs. away from a solid wall) was also recorded.

For each night of trapping in each block, we recorded the number and location of traps set, rats trapped, and traps sprung for other causes (e.g., traps sprung or damaged by people, trapping of non-target species, etc.). The temperature average (°C) and precipitation (mm) for the preceding day were obtained from Environment Canada’s National Climate Data and Information Archive (www.climate.weatheroffice.gc.ca).

After euthanasia, the following measurements were collected from each rat in the field: mass (g), body length (nose to anus, cm), sex, and sexual maturity [animals were considered sexually mature if they had scrotal (vs. inguinal) testes or a perforate (vs. imperforate) vagina [37]]. Rats were identified to species based on external morphology [38] and each rat was palpated over the lumbar spine and pelvic bones and assigned a body condition score based on the scale developed by Hickman and Swan [26]. The presence and number of bite wounds in the skin was recorded, as was the date and location (block and trap) where each rat was trapped.

Rat carcasses frozen at –30°C and shipped to the Animal Health Centre, British Columbia Ministry of Agriculture, Abbotsford, where they were thawed and underwent a standardized necropsy and tissue collection protocol. During the necropsy, sex and sexual maturity were confirmed. When there was a conflict in the assessment of sex or maturity between the field and laboratory researchers, sex and maturity were recorded as ‘undetermined.’ For females, the uterus was examined for pregnancy or placental scars and the mammary chain for evidence of lactation (well developed mammary gland tissue and/or bare, prominent mammae) [37]. A female was considered parous if she was pregnant, had placental scars, and/or was lactating. Embryos were counted when present. Volume of subcutaneous and visceral fat were assessed and used to rate rats on a three point ‘fat scale’ generated for the purposes of this study: poor condition (score of 0) = minimal to no visible internal fat; moderate condition (score of 1) = moderate internal fat; good condition (score of 2) = abundant internal fat. Fat score for an individual animal was arrived at via consensus among at least 2 team members until all members were comfortable using the scale and a subjective degree of consistency had been achieved, at which point, scores were assigned by the individual team member performing the necropsy.

### Data Analysis

All statistical analyses were conducted using R (R Development Core Team, Vienna, Austria) and an alpha level of 0.05 was used to determine statistical significance. Packages utilized in R included ‘pscl’ for zero-inflated negative binomial models, ‘lme4’ for generalized linear mixed models, and ‘survival’ for Cox proportional-hazards models. For all models, individuals with missing values for any of the variables under consideration were excluded. For multivariate models, the goal was to construct the most parsimonious model that predicted the outcome of interest. For each multivariate model, the final model was arrived at by manual stepwise regression using Akaike’s Information Criteria (AIC) to compare candidate models (the final model was that with the lowest AIC) [39]. Collinearity among explanatory variables was identified using Spearman’s rank correlation (ρ) [39]. Highly correlated variables (ρ >0.8) were modeled separately [39]. For each final model, appropriate diagnostics were performed based on the model form to ensure that underlying statistical assumptions were met [39], [40].

#### Population density and distribution

A trap success index was used to measure relative rat abundance in each block [35]–[37]. For each block, this index was calculated by dividing the total number of rats caught by the total trap effort (# traps set×#nights) adjusted according to the method described by Nelson and Clark [41]. This method involves subtracting half a trapping unit from the total trap effort for each trap spring for any cause (e.g., trapping of a rat, trapping of a non-target species, tripping of a trap). This adjustment was considered particularly important because of the marked variation in rat abundance among blocks, and because of the frequency with which traps were sprung by non-target species and by members of the public, both of which can significantly bias trap success indices if they are not taken into account [41]. Because each block under study was roughly the same area (1.2 ha), and because the home range of rats in urban centers is thought to be limited to a city block [42], [43], our trap success indices could be considered to approximate relative population density [35], further increasing comparability. Limited movement of rats among blocks also reduces the possibility of rats migrating into or out of blocks as a result of trapping activities [42], particularly given the relatively short trapping period. Relative abundance was not calculated at the port site because it was not possible to trap rats in this location in the uniform and systematic manor employed in the city blocks.

Variation in the number and location of rats trapped across the study area was visualized spatially in ArcGIS, and Getis-Ord Gi* was used to identify clusters of high and low values for number of rats trapped using the trap as the unit of analysis, the number of rats trapped in that trap as the outcome, a fixed distance band of 148 m (the average length of an alley in the study area), and a Manhattan distance method.

#### Seasonality

In order to determine whether relative rat abundance varied according to season, a zero-inflated negative binomial model was used to evaluate the relationship between trap success in a block (with the block being the unit of analysis) and the season in which trapping took place (September – November = fall, December – February = winter, March – May = spring, June – August = summer).

In order to detect seasonality in population structure, bivariate logistic or linear geneneralized linear mixed models (GLMMs) were used to examine the relationship between sexual maturity (immature vs. mature rats), parity, pregnancy/lactation, and number of embryos (outcome variables) and season (explanatory variable), while controlling for clustering by block of origin (i.e., the city block in which the rat was trapped). Note that, for all analyses, parity and pregnancy/lactation were examined among sexually mature females only. Number of embryos was examined among pregnant females only.

#### Body mass

In order to identify the components that could contribute to mass in rats, we used linear regression to examine the relationship between mass (outcome variable) and length, sex, sexual maturity, body condition score, fat score, block of origin, and season of capture (explanatory variables) in a bivariate and multivariate manner. Block of origin was added as a fixed effect and the outcome variable, mass, was natural logarithm-transformed to satisfy model assumptions.

Two methods of assessing nutritional condition were used in this study: palpation [26] and visual assessment of internal fat stores. These measures were compared using Spearman’s rank correlation.

#### Cutaneous bite wounds

Logistic GLMMs (controlling for block of origin) were used to examine the relationship between wound presence (explanatory variable) and mass, length, sex, sexual maturity, fat score, body condition score, pregnancy, lactation, and season of capture (explanatory variables) in a bivariate and multivariate manner.

Among rats with one or more bite wounds, bivariate and multivariate poisson GLMMs were used to examine the relationship between number of bite wound s and the aforementioned explanatory variables.

#### Factors influencing trappability

Given that rats prefer to travel along walls and avoid open spaces [28], we examined the relationship between the number of rats trapped in any one trap (0 vs. >0) and whether that trap was placed against or away from a solid wall using a logistic GLMM (controlling for block of origin).

In order to determine how ambient temperature and precipitation might influence trap success on a given block-night of trapping (one block-night of trapping is trapping in one block for one night), the relationship between trap success on a block-night (explanatory variable - dichotomized into 0 vs. >0) and average temperature and precipitation (explanatory variables) was examined using a logistic GLMM to control for the random effect of both block of origin and trap day (i.e., when the rat was trapped during the 2 week trapping period).

In order to determine how rat demographic characteristics might influence trapability, bivariate and multivariate logistic GLMMs (controlling for block) were used to examine the relationship between mass, length, sex, sexual maturity, fat score, body condition score, presence and number of bite wounds, pregnancy, and lactation (explanatory variables) and trap day (outcome variable). Trap day was dichotomized to day 1 vs. days 2–10 of trapping.

## Results

### Characteristics of Trapped Rats

A total of 725 rats were collected over the course of the project. Of those 685 (94.5%) were Norway rats and 40 (5.5%) were black rats. Thirty-two of 40 black rats (80.0%) were trapped at the port site, while the remainder (n = 8) were trapped in the city blocks. In contrast, 674 of 685 (98.4%) of Norway rats were trapped in the city blocks and only 11 Norway rats (1.6%) were trapped at the port.

Among the Norway rats, 381 (56.3%) were male and 397 (63.9%) were sexually mature (sex and maturity could not be determined for 8 and 64 rats, respectively). Median mass and length were 142.2 g (range = 20.0–466.2 g) and 17.5 cm (range = 8.5–26.0 cm), respectively. Median body condition on palpation was 2.5/5 and median fat score was 1/2. Bite wounds were present in 169 (24.7%) rats. Among rats with bite wounds, the median number of wounds was 2 (range 1–15). Among sexually mature females (n = 155), 97 (62.6%) were parous, 32 (20.6%) were visibly pregnant, 78 (50.3%) were lactating. Among pregnant females the median number of embryos was 9 (range = 1–15).

Among the black rats 19 (47.5%) were male and 21 (61.8%) were mature (sexual maturity could not be determined for 6 rats). Median mass and length were 76.4 g (range = 24.4–259.8 g) and 13.8 cm (range = 8.5–21.0 cm), respectively. Median body condition on palpation was 2/5 and median fat score was 0/2. Bite wounds were present in 8 (20%) of rats. Among rats with bite wounds, the median number of wounds was 1 (range = 1–3). Among sexually mature females (n = 11), 2 (18.2%) were parous, 1 (9.1%) was pregnant, and 1 (9.1%) was lactating. The pregnant female had 6 embryos.

For subsequent analyses, rats trapped at the port (n = 43, 32 black rats and 11 Norway rats) were excluded because the port trapping scheme was more opportunistic than systematic, and therefore not comparable to that undertaken in the city blocks. Additionally, given that only 8 black rats were trapped outside the port, and given that the ecology of black and Norway rats is known to differ, only Norway rats trapped outside the port (n = 674) were included for further consideration. Henceforth, Norway rats trapped in the city blocks will be referred to as ‘rats.’

### Population Density and Distribution

**Figure 1:**
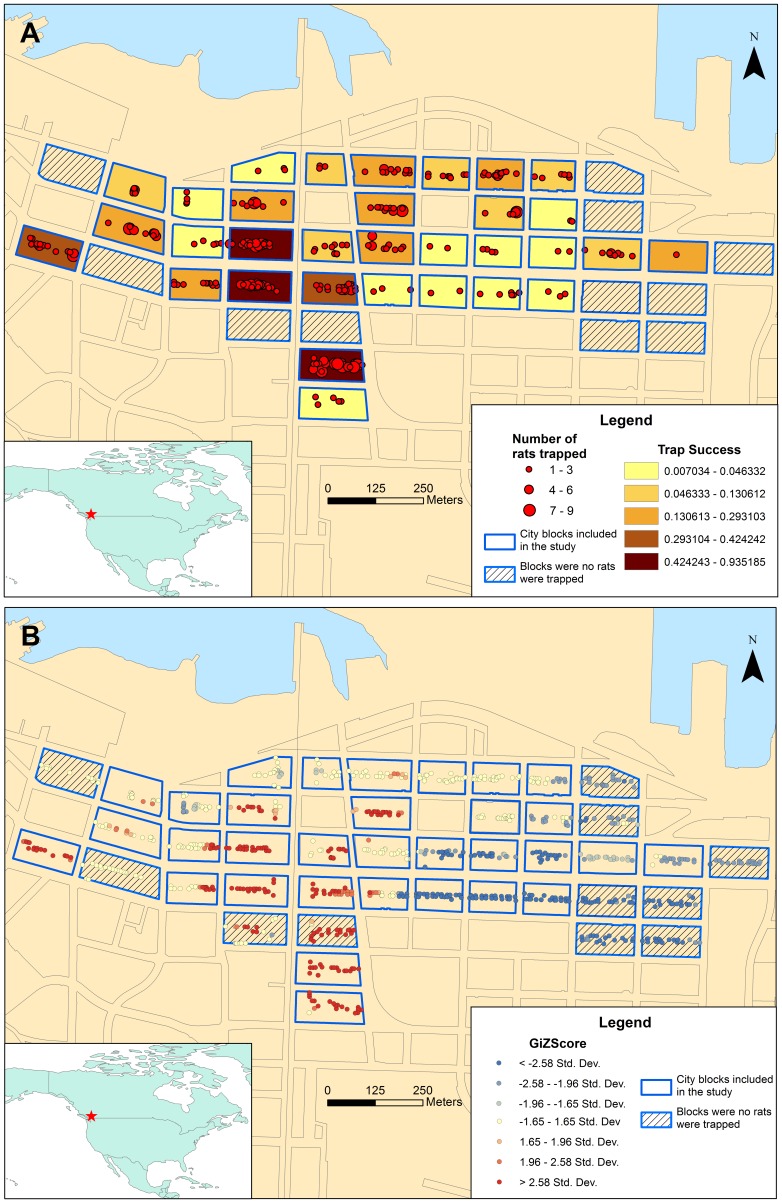
a. Spatial distribution of trapped Norway rats (*Rattus norvegicus*) and trap success in each city block. b. Spatial clusters of relatively high and low rat abundance on Getis-Ord GI* analysis. For the GiZScore, a high z-score indicates spatial clustering of high values and a low z-score indicates spatial clustering of low values. A z-score near zero indicates no significant clustering.

Relative rat abundance varied remarkably among blocks, with trap success ranging from 0 to 0.94 (median 0.04) and geographic clusters of high and low rat abundance were observed (Fig. 1).

### Seasonality

Of the 43 blocks included in the study, 9 were trapped in the fall, 10 in the winter, 14 in the spring and 12 in the summer. There was no significant association between season and rat abundance.

**Figure 2:**
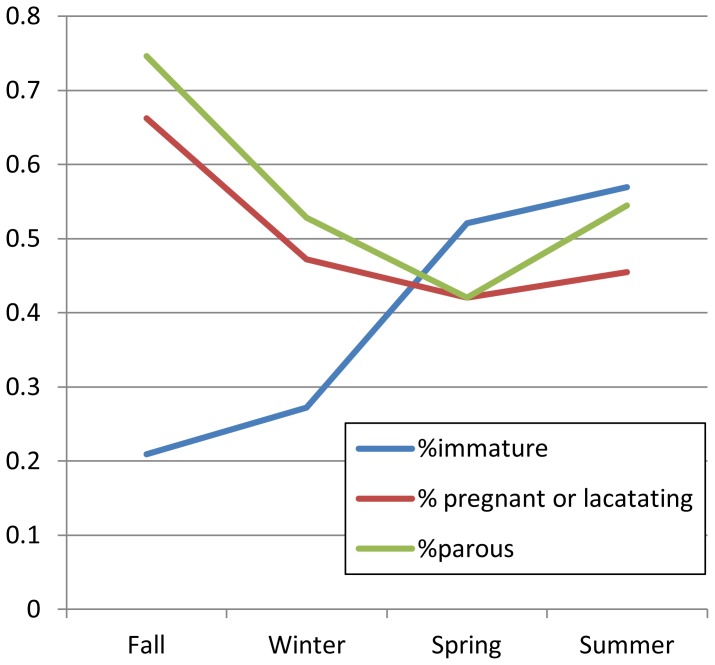
Seasonal variation in the proportion of immature, parous, and pregnant/lactating Norway rats (*Rattus norvegicus*).

Pregnant and lactating rats, as well as sexually immature rats, were found throughout the year, suggesting that reproduction and juvenile recruitment occur year round. However, there was temporal variation in the proportion of the population composed of pregnant/lactating and sexually immature rats. The proportion of immature rats in the population was lowest in the fall, increasing through the winter, spring, and summer (Fig. 2). The proportion of pregnant/lactating rats was highest in the fall, decreasing through the winter and spring before increasing again towards the summer. Parity closely mirrored pregnancy, with the proportion of parous females also being highest in the fall and decreasing through the winter and spring before increasing in the summer.

After controlling for the effect of block, compared to the fall, the odds a rat being sexually immature were 4 times greater in the spring (OR = 3.68, 95% CI = 1.17–11.56) and 5 times greater in the summer (OR = 5.03, 95% CI = 1.17–21.44). There was no significant difference between the fall and winter. The odds of a sexually mature female being pregnant or lactating in the spring was 1/4 that of the fall (OR = 0.28, 95% CI = 0.10–0.82). There was no significant difference in the proportion of pregnant and lactating sexually mature females in the fall compared to summer or winter. The odds of an adult female being parous in the spring was 1/5 that of the fall (OR = 0.20, 95% CI = 0.07–0.59). There was no significant difference in the proportion of parous females in the fall compared to summer or winter. There was no association between season and number of embryos in pregnant females.

### Body Mass

On bivariate analyses, increased log mass was associated with male sex (β = 0.16, 95% CI = 0.05–0.28), sexual maturity (β = 1.33, 95% CI = 1.27–1.40), increased fat score (β = 0.69, 95% CI = 0.64–0.74), increased body condition score (β = 0.23, 95% CI = 0.15–0.30), increased length (β = 0.18, 95% CI = 0.17–0.18), season and block of origin (data not shown). There was no significant association with body condition score.

Maturity and length were highly correlated (ρ = 0.81) and were modeled separately. The final multivariate model included length (β = 0.17, 95% CI = 0.16–0.17), fat score (β = 0.05, 95% CI = 0.02–0.07), and block of origin [for example, rats in block 31 weighed less than those in block 6 (β = −0.19, 95% CI = −0.09– −0.29) while those in block 19 weighed more than those in block 6 (β = 0.06, 95% CI = 0.01–0.12)].

Body condition score on palpation has a significant but weak correlation with fat score as assessed through post-mortem examination (ρ = 0.22, P<0.001).

### Cutaneous Bite Wounds

Among the 674 rats, 166 (24.6%) had bite wounds. On bivariate analysis wound presence was associated with male sex (OR = 1.70, 95% CI = 1.15–2.49), sexual maturity (OR = 11.58, 95% CI = 6.10–22.00), increased mass (OR = 1.14, 95% CI = 1.11–1.16 per 10 g), increased length (OR = 1.46, 95% CI = 1.36–1.58), and increased fat score (OR = 3.11, 95% CI = 2.37–4.10). Among mature females, wound presence was associated with pregnancy (OR = 3.20, 95% CI = 1.40–7.30) and lactation (OR = 2.96, 95% CI = 1.39–6.29). There was no significant association with season or body condition score.

Among the 166 rats with bite wounds, 82 (49.4%) had 1 wound, 31 (18.7%) had 2 wounds, 18 (10.8%) had 3 wounds, 15 (9.0%) had 4 wounds, 8 (4.8%) had 5 wounds, 7 (4.2%) has 6 wounds, 4 (2.4%) had 7 wounds, and 1 had 15 wounds. The rat with 15 wounds was considered an outlier and excluded from the analysis. On bivariate analysis, increased wound number was associated with season (RR = 1.20, 95% CI = 0.91–1.55 for spring vs. fall, RR = 0.94, 95% CI = 0.62–1.43 for summer vs. fall, RR = 1.54, 95% CI = 1.18–2.02 for winter vs. fall), increased mass (RR = 1.02, 95% CI = 1.01–1.03 per 10 g), increased length (RR = 1.06, 95% CI = 1.02–1. 10), and increased fat score (RR = 1.18, 95% CI = 1.01–1.37) among rats with at least one bite wound. There was no significant association with sex, sexual maturity, body condition score, pregnancy, or lactation.

The final multivariate model for wound presence included sex (OR = 1.75, 95% CI = 1.09–2.81 for males vs. females), length (OR = 1.50, 95% CI = 1.38–1.63), and season (OR = 7.40, 95% CI = 2.62–20.95 for spring vs. fall, OR = 7.30, 95% CI = 3.52–15.15 for summer vs. fall; OR = 2.31, 95% CI = 0.92–5.79 for winter vs. fall). The final multivariate model for wound number included sex (RR = 1.24, 95% CI = 0.98–1.58 for males vs. females), mass (RR = 1.02, 95% CI = 1.02–1.03 per 10 g), and season (RR = 1.55, 95% CI = 1.35–1.79 for spring vs. fall; RR = 1.07, 95% CI = 0.70–1.66 for summer vs. fall, RR = 1.54, 95% CI = 1.17–2.02, 0.39–3.93 for winter vs. fall). Note that the following variables were highly correlated and therefore modeled separately: maturity and length (ρ = 0.81), mass and length (ρ = 0.97), mass and maturity (ρ = 0.80), wound presence and wound number (ρ = 0.99).

### Factors Influencing Trappability

Of the 878 traps placed over the course of the project, no rats were trapped in 612 of the trap locations (69.7%). For the remaining 266 trap locations where rats were caught, the median number of rats trapped was 2 (range = 1–9). Five hundred ninety seven (68.0%) of the traps were placed immediately adjacent to a solid wall, while 281 (32%) were not. Trap placement was not significantly associated with the number of rats caught.

There were a total of 354 block-nights of trapping. No rats were caught on 186 (52.5%) of those block-nights. There was no association between trap success on any given block-night (0 vs. >0) and temperature or precipitation.

There were up to 12 days of trapping in a trapping period and we recorded the day of trapping within a trapping period for 649 rat captures. Of those, 144 (22.2%) rats were trapped on the first day of trapping, 95 (14.6%) on day 2, 77 (11.9%) on day 3, 74 (11.4%) on day 4, 66 (10.2%) on day 5, 83 (12.8%) on day 6, 60 (9.2%) on day 7, 44 (6.8%) on day 8, 4 (0.6%) on day 9, and 2 (0.3%) on day 10.

On bivariate analysis, the odds of being trapped on the first day of trapping (vs. subsequent days) was associated with increased mass (OR = 1.03, 95% CI = 1.01–1.05 per 10 g), increased length (OR = 1.11, 95% CI = 1.05–1.17), maturity (OR = 2.29, 95% CI = 1.43–3.66), and the presence of skin wounds (OR = 1.60, 95% CI = 1.03–2.50). There was no significant association with sex, body condition score, fat score, wound number, pregnancy, or lactation.

The final model contained only length, although the bivariate models containing maturity and mass were roughly equivalent. Note that maturity, length, and mass were highly correlated (ρ ≥0.08) and therefore modeled separately.

## Discussion

The results of this study show that urban rat populations have a variety of ecologic features that should be taken into account when studying RAZ.

For example, our data show that that Norway rat population density varies remarkably over a short geographic distance. Not only did density vary among adjacent city blocks, but rats appeared to be distributed non-uniformly along the length of the alley. This heterogeneous distribution is presumably a result of variation in the microenvironment of a city block, particularly as it pertains to resource availability [44], [45]. Given that urban rats are territorial and have a small home range [28], [42], small spatial variations in the abundance of suitable food and harborage could create marked variation in carrying capacity across the urban landscape [21].

The heterogeneous distribution of rats across the urban landscape may, in part, be responsible for observed spatial variations in the presence and prevalence of RAZ [46]. This, in turn, suggests that clustering of rats by city block, or whatever unit best approximates the colony, is an important consideration when designing sampling and analytic protocols for research. For example, it suggests that intensive sampling of rats across a study area is likely necessary in order to achieve adequate representation of populations of interest. This approach is in contrast to many past studies of RAZ, which appear to use less rigorous and/or more opportunistic sampling methods [10], [11], [20], [23], [47]. It also suggests that it is important to control for geographic clustering of rat populations during any data analysis, which does not appear to occur in most studies of rats and their pathogens.

In contrast to observed spatial variation, relative rat abundance did not vary significantly with season. Additionally, pregnant/lactating, parous, and immature animals were present throughout the year, which suggests a lack of strict reproductive seasonality. However, relative population composition did appear to vary significantly among seasons. Specifically, the proportion of mature females that were parous or pregnant/lactating was higher in the summer and fall and lower in the winter and spring, while the proportion of sexually immature rats in the population was lowest in the fall and higher in the winter, spring, and summer. Seasonal variations in reproduction and population demography could influence the ecology of RAZ in a number of different ways. For example, behavioural changes related to mating could influence pathogen transmission, while seasonal changes in the relative proportion of immature vs. mature animals could alter pathogen prevalence [17], [48], and thus the risk of pathogen transmission to people. For example, infection with a some of RAZ, including *Leptospira* spp. [13], [49] and Seoul hantavirus [18], [29], is more common in older rats, suggesting that juvenile recruitment might dilute the prevalence of these pathogens in certain seasons.

Many rodents demonstrate seasonal variations in reproduction, population density, and demography, including several species of bandicoots (*Bandicota* spp.), mice (*Mus* pp), and rats (*Rattus* spp.) [37]. However, there has been considerable conflict among studies regarding if and how urban rat populations are influenced by season [21], [50], and it remains unclear to what degree these discrepancies are a result of methodological inconsistencies vs. true geographic or temporal variation. Given that our findings are based on a single year of study, we cannot definitively conclude that the rat populations under study demonstrate reproductive seasonality [51]. However, it appears prudent to account for season in studies of urban rats and RAZ.

Body mass in rats is often used as a proxy for age in rats, but research has shown this relationship is only approximate and often inaccurate, especially for the youngest and oldest rats in a population [15]. Additionally, previous studies have found significant variation in the growth rate of rats among different habitats, which has been attributed to some combination of genetic heterogeneity and variation in resource availability among different locations [24], [25], [52]. The results of our study, particularly the relationship between mass and length, as well as sexual maturity, support the conclusion that, although age is important determinant of mass, a number of factors independent of age (such as body fat and block of origin) can also influence mass.

Similarly, the presence and number of bite wounds were associated with a variety of demographic factors, such as sex, mass/length, and season. Specifically, bite wounds appear to be most common in longer/heavier rats and male rats, which is consistent with past behavioral studies that suggest that intra-specific aggression is most common among mature males [28]. The association between bite wounds and season, while controlling for mass/length, suggests that there might be seasonal variations in the incidence of intra-specific aggression. Past research has suggested that aggression among male rats is often related to competition for oestrus females [15]; however, the temporal relationship between wounding and pregnancy was less clear in our data. It is interesting to note that in other rodent species, intra-specific aggression also appears to be more common in older males and in certain seasons, although this may vary according to species and location [53], [54]. As with rats [29], [55], intra-specific aggression among males appears to play a role in pathogen transmission among other rodent species [53], [54].

It is of note that the relationship between mass, wounding, and other demographic and environmental factors varied depending on whether they were included in bivariate or multivariate models. This suggests that mass and wounding are complex variables, and that this complexity should be taken into account when using these variables in epidemiologic and ecologic studies. Much of the past research on RAZ has been based solely on biviarate modeling techniques [19], [22]. However, given that ecologic factors have the potential to confound one another, as well as the probability of infection with a zoonotic pathogen, it seems prudent to use more complex statistical methods in the future (e.g., multivariate models) to avoid the identification of erroneous associations.

In this study, we used two different methods to assess nutritional condition. These included a published body condition scoring system utilizing external palpation of the rat’s body [26] and a fat store system (generated for this study) using visual assessment of internal fat stores at necropsy. Scores generated using these two methods were only weakly correlated, and while fat score was associated with a number of different variables mentioned above, far fewer variables were associated with body condition score. Overall, we believe fat score to be the superior variable as it is the most direct reflection of nutritional condition and we found it easier to asses in consistent manner. We are not certain why the body condition scoring system was not successful in our study, but it could be related to the fact that this system was developed for laboratory animals (vs. wild rats, who are generally leaner) or because of our relative inexperience in using this method.

This study employed a trap-removal methodology, which has been used commonly in the past for the study of rats and RAZ [36], [50], [56]–[58]. However, one of the issues common to all trapping-based studies is that it can be difficult to distinguish population characteristics from factors that affect trapability. In our study, we were concerned that trap placement and weather (temperature or precipitation) might affect our ability to trap rats. However, we found that none of these variables were significantly associated with trap success. It was interesting that the majority of rats were caught in the first few days of trapping. Indeed, the number of rats trapped dropped remarkably after the first day with 22.2% of all rats trapped on trap day 1 vs. 14.6% on day 2. This number dropped to <1% by trap day 10. This suggests shorter trapping periods (i.e., of 3–4 days in duration) might be sufficient to collect an adequate sample of urban rats.

That being said, it is important to consider that the characteristics of rats entering the traps may change over the course of the trapping period. For example, we found that sexually mature rats, heavier rats, and longer rats were more likely to be trapped on the first trap day vs. subsequent days. Given that many zoonotic pathogens are more common in older animals [4], short trapping periods might be appropriate for detecting RAZ in rat populations. However, more prolonged trapping might be necessary to obtain a representative sample of the population for epidemiologic studies, and to detect certain pathogens that are more common in young rats (e.g., *Clostridium difficile* [59]).

The aforementioned changes in the characteristics of the study population over the trapping period is interesting given that a week of pre-baiting was conducted in advance of active trapping. Pre-baiting is often used in studies of rats and other rodents in order to allow animals to overcome neophobic responses to traps and bait and thus ensure that all members of trappable population are equally likely to be captured once active trapping is initiated [27], [60]–[62]. The fact that older animals appear to have been trapped first might suggest that more mature, and thus more dominant rats were excluding immature and thus more submissive rats from the food resources within the traps (therefore younger rats began entering traps only when the older animals had been removed) [15]. Many studies of urban rats do not appear to employ pre-baiting [8], [22], [30], and it would be interesting to know how this might bias the characteristics of the trapped population.

Although the specific location of a trap within an alley did not appear to greatly affect the trap success for Norway rats, it is difficult to determine if an overall focus on ground-based outdoor trapping influenced our ability to capture black rats. Among the trapped population, Norway rats comprised 95% of the captures with the remainder being black rats. Most of the black rats were captured at the port area, where trapping was predominantly conducted inside structures. In contrast, 98% of the rats trapped in the city blocks were Norway rats, where traps were set on the ground outside of buildings. Norway rats are thought to reside primarily in underground burrows [44], [63], while black rats are more likely to live in human-made structures [63], [64]. Where these two species co-exist they are thought to remain segregated, with the larger and more aggressive Norway rats excluding the smaller and more timid black rats from ground-based resources [28], [65]. For these reasons our trapping methodology in the city blocks may have resulted in underrepresentation of black rats within the study area. That being said, in many cities, Norway rats are thought to have displaced black rats completely [63] so it is possible that black rats are actually rare within the majority of the study area. It should also be noted that rats in this study were identified to species based on external morphologic characteristics; therefore ‘cryptic’ *Rattus* spp. may have been overlooked. These cryptic species are morphologically indistinguishable from Norway and/or black rats, and therefore can only be recognized using genetic techniques [66].

Overall, the results of this study shed some light on the characteristics of urban rat populations that could influence the ecology and study of RAZ. However, there are still a variety of aspects requiring further elucidation. For example, although we were able to show that rat population density varies by city block, further research will be needed to identify the precise reasons behind this variation. There is some indication that the heterogeneous distribution of rats across the urban landscape is a result of variations in the environment with regard to resource availability [21], [44]. However, rat behaviuor and/or genotype, could also play a role in abundance and distribution [64]. Indeed, information on the genetic structure of rat populations, which has received little attention in the past, has the potential to deepen our understanding of both rat and RAZ ecology. Additionally, although we were able to identify seasonal variation in population structure, we could not account for seasonal variations in rat behaviuor, vector ecology, or rat-human interactions, all of which can influence RAZ ecology [48], [67], [68]. Perhaps most importantly, the use of a trap-removal methodology and the fact that the study took place over one year meant that we were not able to capture a variety of longitudinal changes in rat populations, for example, inter-annual variation and changes due to anthropogenic modification in the urban environment (e.g., urban decay and re-development). Scientists are well aware that long term environmental alterations, such as climate change, are likely to have significant impacts on the ecology of wild animals and their diseases [69]. However, little attention has been paid to the changes that occur in urban ecosystems, which would seem much more dramatic and rapid, particularly from the perspective of urban rats. For this reason we suggest that cities around the world should invest in surveillance and research that aims to understand and monitor local rat populations now and into the future.
